# Murine Endometrial Organoids to Model *Chlamydia* Infection

**DOI:** 10.3389/fcimb.2020.00416

**Published:** 2020-08-14

**Authors:** R. Clayton Bishop, Matteo Boretto, Melanie R. Rutkowski, Hugo Vankelecom, Isabelle Derré

**Affiliations:** ^1^Department of Microbiology, Immunology, and Cancer Biology, University of Virginia, Charlottesville, VA, United States; ^2^Unit of Stem Cell Research, Cluster of Stem Cell and Developmental Biology, Department of Development and Regenerations, University of Leuven, Leuven, Belgium

**Keywords:** *Chlamydia*, endometrial organoids, 3D culture, developmental cycle, inclusion membrane proteins

## Abstract

The obligate intracellular bacterium *Chlamydia trachomatis* is the leading cause of bacterial sexually transmitted infections. Once internalized in host cells, *C. trachomatis* undergoes a biphasic developmental cycle within a membrane-bound compartment, known as the inclusion. Successful establishment of the intracellular niche relies on bacterial Type III effector proteins, such as Inc proteins. *In vitro* and *in vivo* systems have contributed to elucidating the intracellular lifestyle of *C. trachomatis*, but additional models combining the archetypal environment of infection with the advantages of *in vitro* systems are needed. Organoids are three-dimensional structures that recapitulate the microanatomy of an organ's epithelial layer, bridging the gap between *in vitro* and *in vivo* systems. Organoids are emerging as relevant model systems to study interactions between bacterial pathogens and their hosts. Here, we took advantage of recently developed murine endometrial organoids (EMOs) and present a *C. trachomatis*-murine EMO infection model system. Confocal microscopy of EMOs infected with fluorescent protein-expressing bacteria revealed that inclusions are formed within the cytosol of epithelial cells. Moreover, infection with a *C. trachomatis* strain that allows for the tracking of RB to EB transition indicated that the bacteria undergo a full developmental cycle, which was confirmed by harvesting infectious bacteria from infected EMOs. Finally, the inducible gene expression and cellular localization of a *Chlamydia* Inc protein within infected EMOs further demonstrated that this model is compatible with the study of Type III secreted effectors. Altogether, we describe a novel and relevant system for the study of *Chlamydia*-host interactions.

## Introduction

*Chlamydia* is the most commonly reported bacterial sexually transmitted disease in the world with rates continuing to increase annually (Centers for Disease Control and Prevention, 2019). The etiological agent*, Chlamydia trachomatis*, is an obligate intracellular bacterium that, after colonization of the lower female reproductive tract, ascends to infect the uterine endometrium and fallopian tubes. Currently, there is no vaccine available against *Chlamydia* (Phillips et al., 2019). Although antibiotics can be effective against *C. trachomatis* infections, 70–80% of infections in women are asymptomatic and thus left untreated (Geisler, 2010; Malhotra et al., 2013). Untreated infections can lead to pelvic inflammatory disease, ectopic pregnancy, and infertility (Haggerty et al., 2010). Following entry into a host epithelial cell, *Chlamydia* resides within a membrane-bound compartment, known as the inclusion (Gitsels et al., 2019). Within the inclusion, *Chlamydia* undergoes a biphasic developmental cycle alternating between two distinct bacterial forms (Moulder, 1991; Abdelrahman and Belland, 2005). Infection begins with the infectious, but non-replicative elementary body (EB). The EB-containing inclusion traffics toward the nucleus, and EBs transition to the replicative, but non-infectious form (reticulate body, RB). After multiple rounds of replication and ~24–36 h post-bacterial entry, the RBs asynchronously transition back to the EB form. After 2–3 days of infection, EBs are released from the host cell following cell lysis or by extrusion (Hybiske and Stephens, 2007; Zuck et al., 2016).

As an intracellular bacterium, *Chlamydia* must remodel the membrane of the inclusion and manipulate its host cell to establish its replication niche. This process is mediated via a number of bacterially-encoded Type III effector proteins (Ferrell and Fields, 2016; Bugalhão and Mota, 2019). Some of these effector proteins, known as inclusion membrane proteins (Inc), are translocated to and embedded within the inclusion membrane to mediate interaction between the inclusion and the host (Dehoux et al., 2011; Lutter et al., 2012; Moore and Ouellette, 2014; Bugalhão and Mota, 2019; Gitsels et al., 2019).

Much of what is known regarding *Chlamydia*'s mechanisms to manipulate host cells comes from *in vitro* studies using cancer-derived cell lines, such as HeLa cells, which provide ease of maintenance and genetic manipulation, but lack much of the relevance of an *in vivo* environment (Dolat and Valdivia, 2019). Beyond cancer-derived cell lines, immortalized and polarized cell lines of reproductive tissues have broadened the relevance of *in vitro* systems (Igietseme et al., 1994; Guseva et al., 2005, 2007; Moore et al., 2008; King et al., 2009), and primary cells have provided systems with minimal artificial manipulation of cells (Prozialeck et al., 2002; Roth et al., 2010; Zadora et al., 2019; Maffei et al., 2020). Further, the use of three-dimensional (3D) and multi-cell type models have allowed for incorporating aspects of cell-cell interactions and tissue architecture (Nogueira et al., 2017; Edwards et al., 2019). Finally, whole tissue or *ex vivo* models are among the most relevant established systems, as they exhibit a more complete environment of infection (Hutchinson et al., 1979; Cooper et al., 1990; Roth et al., 2010; Jerchel et al., 2012; Kessler et al., 2012). However, they cannot be maintained long-term and are not easily accessible. Murine models to study pathogenesis and host immune response associated with *Chlamydia* infection are also commonly used, but the study of specific molecular mechanisms involved is limited (De Clercq et al., 2013). While each of the above-mentioned systems has its benefits, additional model systems that better combine the advantages of *in vitro* systems and *in vivo* models would be valuable to gain a better understanding of the molecular mechanisms underlying the interaction of *C. trachomatis* with its host cell.

Organoids present an *ex vivo* model system that bridges the gap between artificial *in vitro* studies and less tractable *in vivo* systems. Under niche-mimicking culture conditions, adult stem cells self-organize into 3D near-physiological structures termed organoids (Shamir and Ewald, 2014). The stemness of organoids results in a self-renewing system that can be cultivated indefinitely. Organoids have been generated from various tissue types, most notably the intestinal epithelium, and were originally used as a tool to study mammalian tissue biology and diseases (Dutta et al., 2017). In the last 6 years, the use of intestinal organoids was extended to the study of bacterial infection with *Helicobacter pylori, Escherichia coli, Salmonella*, and *Clostridium difficile* (Zhang et al., 2014; Forbester et al., 2015; Leslie et al., 2015; Bartfeld, 2016; In et al., 2016; Tao et al., 2016; Dutta and Clevers, 2017; Dutta et al., 2017; Pompaiah and Bartfeld, 2017; Pleguezuelos-Manzano et al., 2020).

Two female reproductive tract organoid models were recently developed and show promise for the study of sexually transmitted infections (STIs). First, human fallopian tube organoids were used to model chronic *C. trachomatis* infection (Kessler et al., 2015, 2019). Second, endometrial organoids (EMOs), which recapitulate the epithelial layer of the endometrium, defined as the innermost layer of the uterus, were established from both murine and human tissues (Boretto et al., 2017, 2019; Turco et al., 2017). EMOs are generated by culturing stem cells found within endometrial glands, which are proposed to be responsible for regenerating the epithelial cell layer of the endometrium (Gargett et al., 2016; Boretto et al., 2017; Turco et al., 2017; Cousins et al., 2018). EMOs contain both the ciliated epithelial cells and secretory cells present in the endometrial epithelial layer. Additionally, they maintain accurate cell polarity, cell-cell junctions, mucus production, and sex hormone response (Boretto et al., 2017; Turco et al., 2017). Despite the great potential of EMOs to increase the relevance of the study of STIs, they have not been used in the context of infections with pathogens.

Here, we describe a novel murine EMO model system to study *Chlamydia* infection. We demonstrate that *C. trachomatis* successfully infects and completes its developmental cycle within murine EMOs. In addition, we show that this system is compatible with inducible gene expression and detection of tagged *Chlamydia* Inc proteins by immunofluorescence microscopy. Together, our data show that the *C. trachomatis*-EMO model system has the potential to preserve the tools of *in vitro* models while contributing aspects of the cellular environment of *in vivo* systems.

## Materials and Methods

### Ethics Statement

All genetic manipulations and containment work were approved by the UVA Biosafety Committee and are in compliance with the section III-D-1-a of the National Institutes of Health guidelines for research involving recombinant DNA molecules. Mice were not bred for the sole purpose of harvesting endometrial tissues. Endometrial tissues for EMO generation was sourced from otherwise discarded murine reproductive tracts.

### Cell Lines and Bacterial Strains

HeLa cells were obtained from the ATCC (CCL-2) and cultured at 37°C with 5% CO_2_ in high-glucose Dulbecco's modified Eagle's medium (DMEM; Invitrogen) supplemented with 10% heat-inactivated fetal bovine serum (FBS; Invitrogen). *C. trachomatis* Lymphogranuloma venereum, Type II were obtained from ATCC (L2/434/Bu VR-902B). *C. trachomatis* strain expressing mCherry alone and the RB-to-EB transition fluorescent reporter strain were described previously (Cortina et al., 2019). The *C. trachomatis* strain expressing mCherry constitutively and IncV-3xFLAG under the control of the anhydrotetracycline inducible promoter was described previously (Stanhope et al., 2017). *C. trachomatis* propagation and HeLa cell infection was performed as previously described (Derré et al., 2007).

### Murine Endometrial Organoid Culture

The method for generating murine endometrial organoids was adapted from Boretto et al. as follows (Boretto et al., 2017). Uterine horns were isolated from otherwise discarded genital tract tissue from naïve 6–8 week-old female C57BL/6J mice, bisected, and minced. Tissue fragments were incubated in 30 mM ethylenediaminetetraacetic acid (EDTA) in PBS for 1 h at 37°C followed by mechanical dissociation in cold PBS. Dissociated tissue and supernatant were passed through a 70 μm cell strainer (Corning) to isolate endometrial glands and supplemented with 10% heat inactivated FBS. Isolated glands in PBS/FBS were centrifuged at 230 g for 5 min, and the pellet was resuspended in DMEM. The sample was centrifuged at 230 g for 5 min, and the pellet was resuspended in mixture of 70% growth factor reduced Matrigel (Corning) in DMEM. Ten to twenty microliters drops of the suspension were plated onto pre-warmed non-treated 24 well plates (1–2 drops per well) and incubated at 37°C for ~20–30 min to allow for Matrigel solidification. Mouse IntestiCult^TM^ organoid growth medium (STEMCELL Technologies) supplemented with 1X penicillin-streptomycin-glutamine (PSQ; Gibco) was added to cover the Matrigel drops. Cultures were maintained at 37°C with 5% CO_2_, and culture medium was changed every 2 days. EMOs were visible within 2–5 days.

EMOs were passaged every 7–14 days. Culture medium was removed from the wells, and TrypLE^TM^ Express Enzyme (1X) without phenol red (Gibco) was added for 15 min at 37°C. TrypLE^TM^ was inactivated by addition of DMEM. EMOs were collected and centrifuged at 230 g for 5 min at 4°C. The pellet was resuspended in DMEM and subjected to mechanical disruption to fully dissociate EMOs into single cells. Dissociated cells were centrifuged at 230 g for 5 min at 4°C, and the pellet was resuspended in 70% Matrigel in DMEM and plated as described above. Culture medium supplemented with 1X PSQ and 10 μM Y-27632 (ROCK inhibitor; Millipore Sigma) was added. Culture medium containing ROCK inhibitor was used for the first subsequent medium change, but removed for additional medium changes.

### Infection of Murine Endometrial Organoids

One day prior to infection, culture medium was replaced with medium free of PSQ. At the time of infection, culture medium was removed, and cold PBS was added to dissolve Matrigel, followed by vigorous pipetting to break apart EMOs. The fragmented EMOs were incubated in DMEM for 15 min at 37°C for 5% CO_2_, followed by centrifugation at 290 g for 5 min. The pellet of ~50–100 fragmented EMOs was incubated with ~5 × 10^9^ inclusion forming units (IFU) of the indicated strain of *C. trachomatis* in 100 μl of DMEM for 1 h at 37°C to allow for infection. Matrigel was added to generate a 70% Matrigel mixture which was plated as described above. IntestiCult^TM^ without PSQ or Rock inhibitor was used.

### Immunofluorescence and Confocal Microscopy

Uninfected or infected EMOs embedded in Matrigel were plated in eight well-chambered coverglass wells (Thermo Scientific Nunc). At the indicated times, EMOs were fixed in 1X PBS containing 4% paraformaldehyde for 1 h at room temperature (RT). Antibodies and fluorescent dyes were diluted in PBS containing 0.5% Triton X-100 and 5% bovine serum albumin (BSA). Incubation with primary and secondary antibodies was conducted overnight at 4°C and 1–2 h at RT, respectively. Primary and secondary staining of fixed samples was followed by three washes with 1X PBS.

For Figure 3B, HeLa cells seeded onto glass coverslips were fixed for 20 min in PBS containing 4% paraformaldehyde. Coverslips were stained with Hoechst dye diluted in PBS containing 0.1% Triton X-100 for 1 h at RT, followed by three washes with 1X PBS. Coverslips were mounted with DABCO antifade-containing mounting media.

Samples were imaged with a Nikon epifluorescence microscope or a Leica DMi8 confocal microscope equipped with the Andor iXon ULTRA 888BV EMCCD camera and driven by the IQ software. Where indicated, live samples were imaged in a humidified live cell environmental chamber set at 37°C and 5% CO_2_. Images were processed using the Imaris software (Bitplane, Belfast, United Kingdom).

### Antibodies and Dyes

The following primary antibodies and dyes were used: goat polyclonal anti-*Chlamydia* major outer membrane protein (MOMP; 1:200, Virostat) and mouse monoclonal anti-FLAG (1:1,000, Sigma). The following secondary antibodies were used: Alexa Fluor^TM^ 488-conjugated donkey anti-goat IgG (1:500, Molecular Probes) and Alexa Fluor^TM^ 488- or 594-conjugated goat anti-mouse IgG (1:500, Molecular Probes). The following dyes were used: CellMask^TM^ Green (1:200, Invitrogen), SYTOX^TM^ Green (1:10,000, Invitrogen), SYTOX^TM^ Blue (1:5,000, Invitrogen), Alexa Fluor^TM^ 594 Phalloidin (1:200, Molecular Probes), Hoechst (1:1,000, Molecular Probes), and TO-PRO^TM^-3 Iodide (1:1,000, Invitrogen).

### Harvest of *C. trachomatis* Infectious Progeny From Infected Endometrial Organoids

EMOs were infected with mCherry-expressing *C. trachomatis* as described above. At 48 hpi (hours post-infection), the culture medium was removed and replaced with cold water to dissolve the Matrigel, followed by vigorous pipetting to break apart and lyse epithelial cells of the EMOs and release the bacteria. Bacteria and unlysed cells were collected and centrifuged at 8,000 rpm for 20 min at 4°C. Pelleted sample was resuspended in cold water, followed by additional vigorous pipetting and centrifugation with the same conditions to ensure that all infected cells were lysed. Pelleted sample was resuspended in RT DMEM supplemented with 10% FBS and plated onto a confluent HeLa cell monolayer, followed by centrifugation at 1,200 rpm for 20 min at RT. HeLa cells were incubated with harvested EBs for 32 h at 37°C in the presence of 5% CO_2_, at which point samples were fixed, stained, and imaged as described above.

### Anhydrotetracycline Induction of IncV-3xFLAG

EMOs were infected with *C. trachomatis* expressing mCherry constitutively and IncV-3xFLAG under the control of the anhydrotetracycline inducible promoter, as described above. At 24 hpi, the culture medium was replaced with culture medium supplemented with 20 ng/ml aTc. Infected EMOs were incubated in the presence of aTc at 37°C with 5% CO_2_ for 6 h, at which point samples were fixed, stained, and imaged as described above.

### Experimental Replication

Images presented in this study are representative of multiple infected EMOs analyzed from at least three independent experiments performed with independent EMO preparations.

## Results

### Generation of Murine Endometrial Organoids

**Figure 1:**
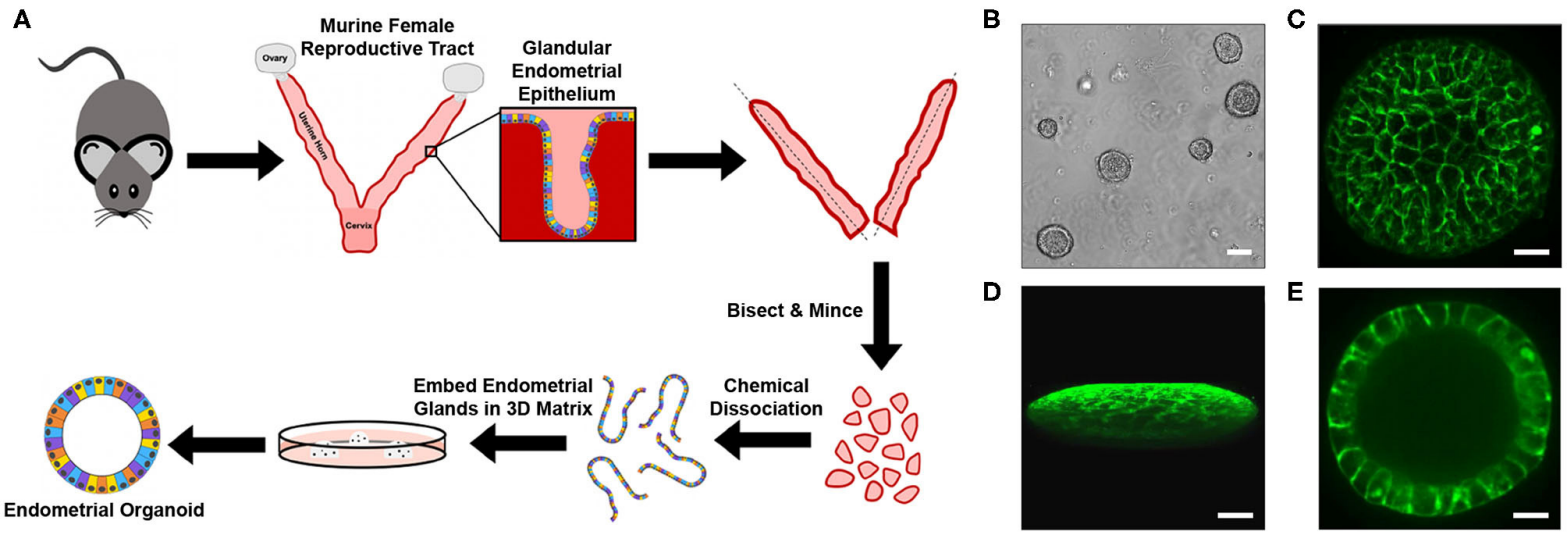
Generation of murine EMOs. **(A)** Cartoon diagram depicting the method used to generate EMOs from female C57BL/6J mice. **(B)** Brightfield image of mature murine EMOs embedded in Matrigel following passage and expansion. Scale bar: 80 μm. **(C–E)** Confocal micrographs of a live EMO stained with CellMask^TM^ Green to mark plasma membranes. A top view of a 3D reconstruction of an EMO **(C)**, a side view of a 3D reconstruction of an EMO **(D)**, and a single plane across the middle of an EMO **(E)** are shown. Scale bar: 50 μm.

Murine endometrial organoids (EMOs) were generated by adaptation of a previously established protocol (Boretto et al., 2017). Uterine horns excised from otherwise discarded genital tract tissues of naïve mice were bisected and minced to expose the endometrium (Figure 1A). Endometrial glands, which contain the stem and progenitor cells that replenish the endometrium during the estrous cycle (Gargett et al., 2016; Cousins et al., 2018), were isolated by chemical dissociation of the tissue, embedded within a 3D extracellular matrix, Matrigel, and cultured in the presence of growth factors. Within ~7 days, mature EMOs were observed (Figure 1B). EMOs were then passaged and expanded every 7–14 days by dissociation into individual cells by trypsin digest and re-embedding in Matrigel. Live confocal imaging of EMOs, stained with CellMask^TM^ to visualize the plasma membrane of individual epithelial cells, revealed the previously observed 3D oblate spheroid structure composed of a single cellular layer surrounding a hollow lumen (Figures 1C–E).

### Infection of Murine Endometrial Organoids With *C. trachomatis*

**Figure 2:**
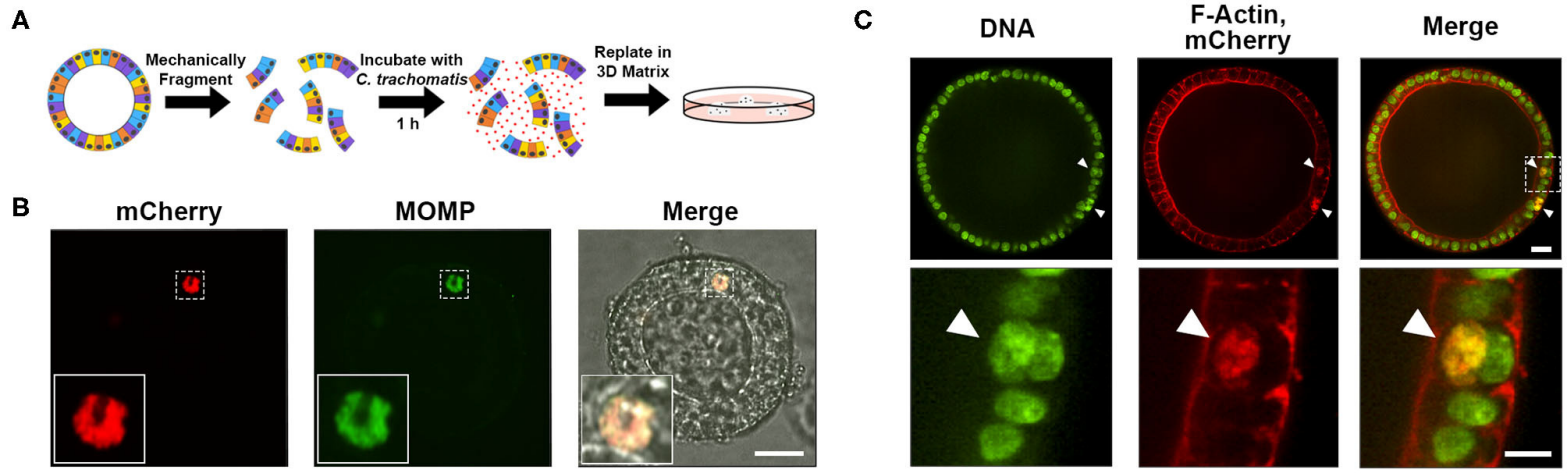
Infection of murine EMOs with *C. trachomatis*. **(A)** Cartoon diagram depicting the method of infecting EMOs with *C. trachomatis* by mechanical fragmentation. **(B)** Confocal micrographs of an EMO infected with mCherry-expressing *C. trachomatis* (red, left panel, mCherry). Infected EMOs were fixed at 32 hpi and immunostained with anti-*C. trachomatis* MOMP (green, middle panel, MOMP). The merge, including a brightfield image, is shown on the right. Higher magnification of the indicated boxed area is shown in the inset of each panel. A single plane across the middle of the inclusion is shown. Scale bar: 40 μm. **(C)** Confocal micrographs of an EMO infected with mCherry-expressing *C. trachomatis* (red, middle panels, mCherry) fixed at 32 hpi and stained with SYTOX^TM^ Green (green, left panels, DNA) and Phalloidin (red, middle panels, F-Actin). The merge is shown on the right. A single plane across the middle of the EMO and inclusions is shown. Top panels: whole EMO. Scale bar: 40 μm. Bottom panels: higher magnification of indicated boxed area. Inclusions are indicated by arrow heads. Scale bar: 20 μm.

As *Chlamydia* encounters the apical surface of the endometrial epithelium during natural infection, one obstacle to EMO infection is the polarization of the epithelial cells with the apical side facing the lumen (Boretto et al., 2017). To circumvent this problem, 3D EMOs were mechanically fragmented prior to incubation with mCherry-expressing *C. trachomatis* for 1 h (Figure 2A). Infected EMO fragments were then embedded in Matrigel and incubated for 32 h to allow for 3D EMOs to reform and the bacteria to replicate. Immunostaining of *C. trachomatis* major outer membrane protein (MOMP) revealed the presence of bacteria-filled inclusions within the cellular layer of EMOs (Figure 2B). To determine the specific localization of inclusions, infected EMOs were fixed and stained with SYTOX^TM^ Green to visualize nuclei (Figure 2C, green) and red fluorescent Phalloidin to visualize cortical actin and microvilli, thereby allowing detection of cell boundaries (Figure 2C, red). Inclusions, which were indicated by mCherry fluorescence and SYTOX^TM^ Green staining, were detected within the cytosol of individual cells. Together, these data indicate that *C. trachomatis* successfully infects murine EMOs and forms inclusions within the cytosol of epithelial cells.

### *C. trachomatis* Developmental Cycle Within Murine Endometrial Organoids

**Figure 3:**
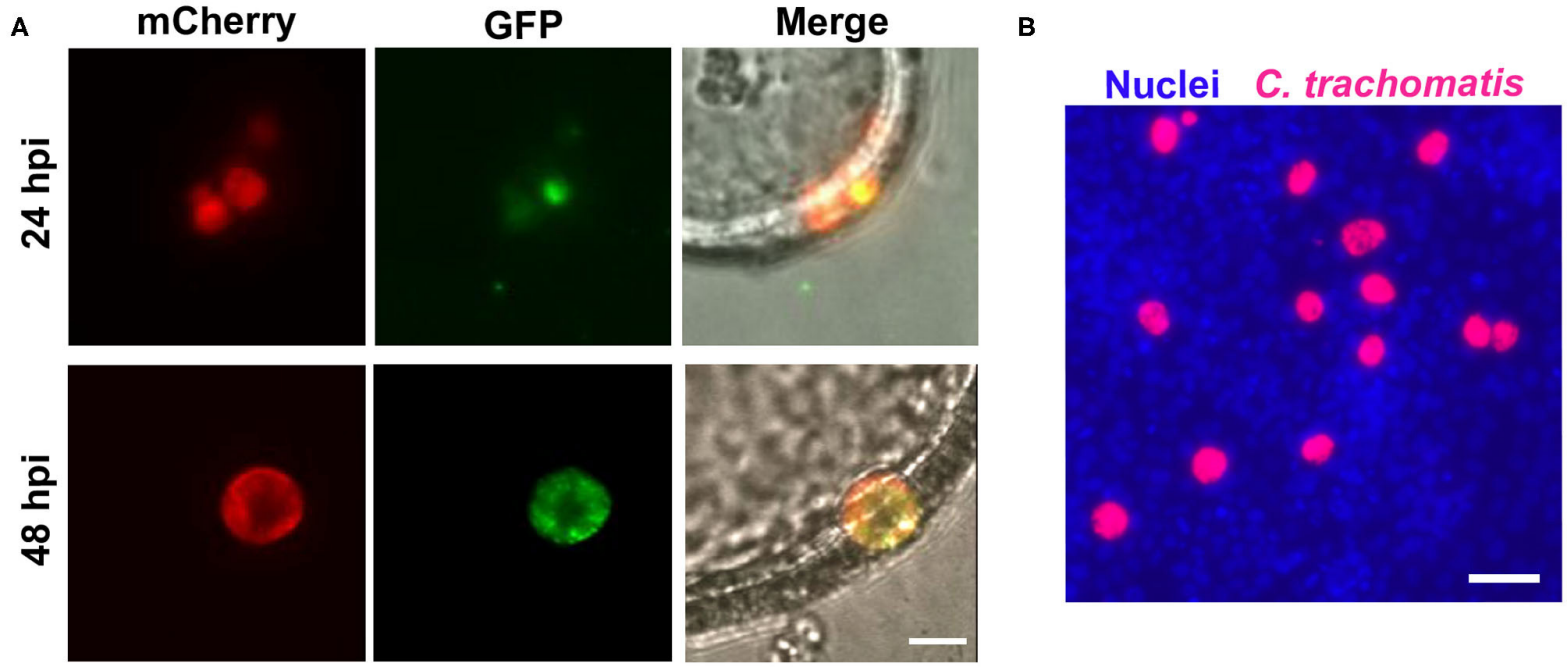
*C. trachomatis* completes a full developmental cycle in murine EMOs. **(A)** Live epifluorescence micrographs of two different EMOs infected with the RB-to-EB reporter strain of *C. trachomatis* for 24 h (top panels) or 48 h (bottom panels). Bacteria at the RB stage expressing mCherry alone are shown in red (left panels, mCherry), and bacteria that have transitioned to EB stage expressing GFP are shown in green (middle panels, GFP). The merge, including a brightfield image, is shown on the right. A single plane across the middle of the EMO and inclusions is shown. Scale bar: 10 μm. **(B)** Immunofluorescence image of a HeLa cell monolayer infected with mCherry-expressing *C. trachomatis* (red) collected from infected EMOs at 48 hpi. At 32 hpi, the infected HeLa cells were fixed and stained with the Hoechst DNA dye (blue). Scale bar: 40 μm.

To determine if *C. trachomatis* underwent a complete developmental cycle within EMOs, we used our fluorescent reporter strain of *C. trachomatis* that allows for tracking of the RB to EB transition (Cortina et al., 2019). This strain expresses the mCherry fluorescent protein under the control of the *groESL* promoter, resulting in constitutive expression throughout the developmental cycle. It also expresses the green fluorescent protein (GFP) under the control of the late *omcA* promoter, which is expressed in the middle of the developmental cycle as bacteria transition from the RB to EB form (Shaw et al., 2000; Belland et al., 2003). At 24 h post-infection (hpi) murine EMOs infected with the RB-to-EB reporter strain displayed inclusions that were positive for mCherry with very few bacteria expressing GFP (Figure 3A, 24 hpi). At 48 hpi, inclusions had grown in size and were filled with GFP-expressing bacteria, indicating that RBs had successfully transitioned to EBs (Figure 3A, 48 hpi). To independently confirm the production of infectious EBs, EMOs were infected with mCherry-expressing *C. trachomatis*, and bacteria harvested from EMOs 48 hpi were used to infect HeLa cells (Figure 3B). The presence of mCherry-positive inclusions within the HeLa cell monolayer indicated that infectious EBs were indeed produced within EMOs. Altogether, these data demonstrate that *C. trachomatis* undergoes a full developmental cycle and generates infectious progeny within murine EMOs.

### Induction of Gene Expression and Inclusion Localization of Inc Protein Within Infected Murine Endometrial Organoids

**Figure 4:**
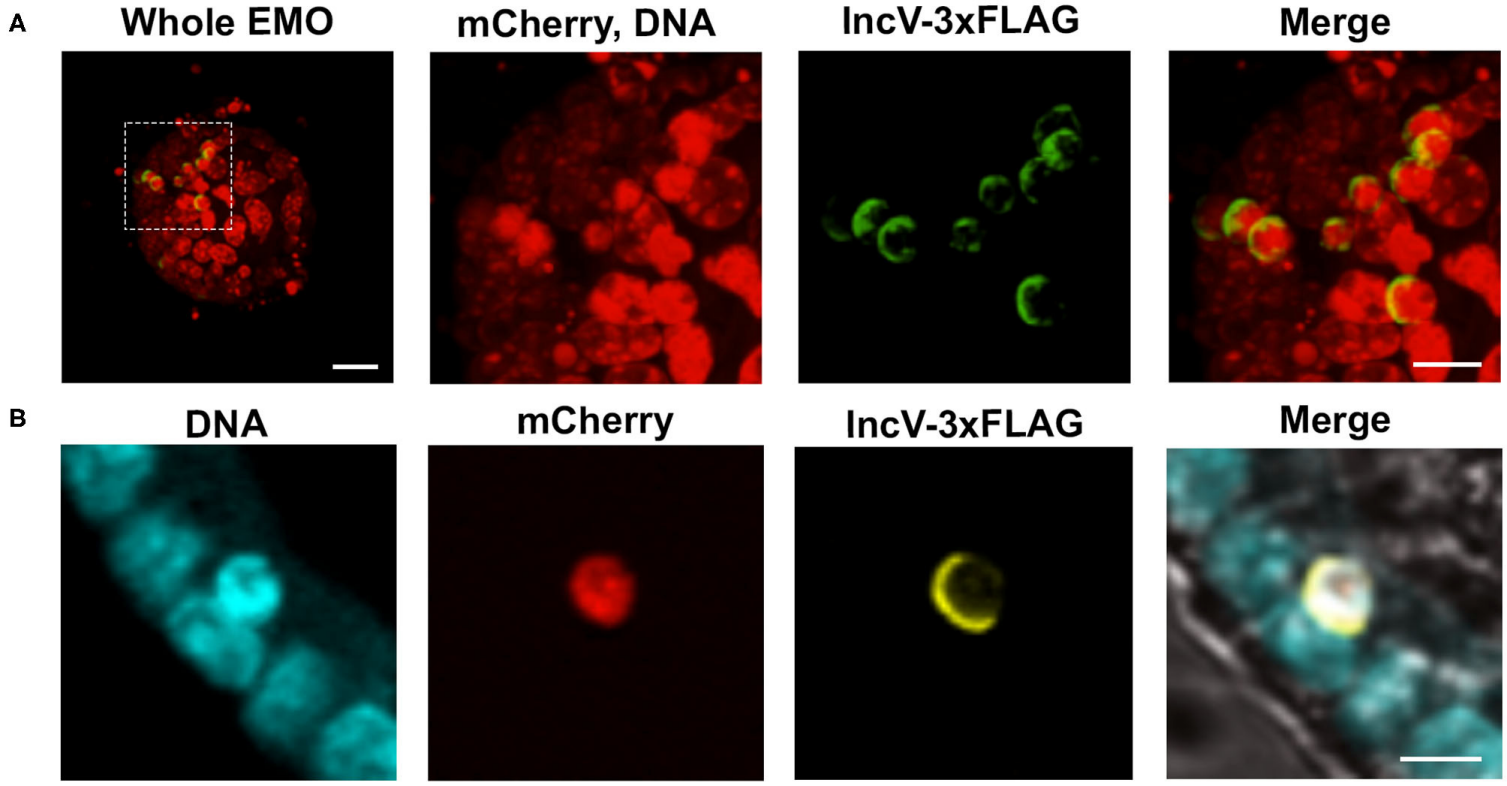
Induction of gene expression and inclusion localization of an Inc protein within infected murine EMOs. **(A)** 3D confocal micrographs of an EMO infected for 30 h with mCherry-expressing *C. trachomatis* constitutively (red) and IncV-3xFLAG under the control of an aTc inducible promoter (green), and stained with TO-PRO^TM^-3 (red, DNA). A low magnification merge image corresponding to the whole EMO is shown on the left (Whole EMO). Scale bar: 40 μm. A high magnification of individual channels and the merge image of the indicated boxed area is shown in the three panels to the right. Scale bar: 20 μm **(B)** Confocal micrographs of an EMO infected for 30 h with *C. trachomatis* expressing mCherry constitutively (red) and IncV-3xFLAG under the control of an aTc inducible promoter (yellow), stained with SYTOX^TM^ Blue (blue, DNA) and antibodies against FLAG (yellow, IncV-3xFLAG). The merge, including a brightfield image, is shown on the right. A single plane across the middle of the EMO and inclusion is shown. Scale bar: 20 μm.

*Chlamydia* relies on inclusion-localized Inc proteins to facilitate direct interaction between host cell proteins and the inclusion membrane (Dehoux et al., 2011; Lutter et al., 2012; Moore and Ouellette, 2014; Bugalhão and Mota, 2019; Gitsels et al., 2019). Strains of *C. trachomatis* expressing tagged Inc proteins under an inducible promoter have become a valuable tool to assess the localization and function of Inc proteins during infection (Agaisse and Derré, 2014; Bauler and Hackstadt, 2014; Mirrashidi et al., 2015; Weber et al., 2015; Han and Derré, 2017; Nguyen et al., 2018). With this system, the expression of the *inc* gene of interest is induced by the addition of anhydrotetracycline (aTc), and the corresponding protein is detected by immunofluorescence microscopy. To test if this system was applicable to EMOs, EMOs were infected with *C. trachomatis* expressing mCherry constitutively and IncV-3xFLAG under the control of the aTc inducible promoter. At 24 hpi, IncV-3xFLAG expression was induced for 6 h followed by fixation and immunostaining with anti-FLAG antibodies. Confocal microscopy revealed that IncV-3xFLAG expression was successfully induced within EMOs. As exemplified by Figure 4A, multiple inclusions within an infected EMO were positive for the IncV-3xFLAG signal. This result was also confirmed by imaging a single Z plane across an inclusion (Figure 4B). Moreover, we note the crescent shape pattern of the IncV signal on one side of the inclusion, which is in agreement with the previously described inclusion localization of IncV in HeLa cells (Stanhope et al., 2017). The ability to induce and detect the expression of IncV-3xFLAG in infected EMOs demonstrates that the EMO model system is compatible with the study of the cellular localization of *Chlamydia* inclusion membrane proteins and, most likely, effector proteins in general.

## Discussion

Much of the current understanding of the molecular processes used by *Chlamydia* to interact with its host cell comes from *in vitro* systems (Dolat and Valdivia, 2019). Although these models are more easily maintained and manipulated than *in vivo* models, they do not fully recapitulate the cell-cell interactions and 3D nature present in the natural environment of the infected tissues. EMOs present an *ex vivo* model system that bridges the gap between less relevant *in vitro* studies and less tractable *in vivo* systems. Our data demonstrate that murine EMOs can serve as a novel model system in which to study *Chlamydia* host-pathogen interactions.

Our methods described here for generating and infecting murine EMOs were developed to be accessible and without the need for specialized equipment. The largest barrier to infection of EMOs with *C. trachomatis* is access to the cellular apical surface. A number of different methods have been developed for bacterial infection of organoids, including generation of 2D organoid monolayers and microinjection of bacteria into organoid lumens (Dutta et al., 2017; Yin and Zhou, 2018). Microinjection is especially appropriate for invasion or early infection studies and offers the advantage of minimally disrupting the integrity of the EMO at the time of infection. It also allows for a tight control of the number of bacteria delivered in the EMO lumen. Another method, which was used here, is infection of mechanically fragmented organoids. This approach leads to a temporary disruption of the 3D architecture of the EMOs and does not allow for a precise control of the MOI, which would preclude it from invasion studies. Nevertheless, the cellular organization of each EMO fragment is preserved and post-invasion stages of the infection can be monitored in the 3D environment of the primary cell epithelium of the resealed EMO. This method is also less technically challenging and does not require any special equipment.

Our analysis of *C. trachomatis* infected EMOs indicated that *Chlamydia* undergoes a productive developmental cycle in EMOs. Beyond 48 h of infection, we observed ruptured inclusions (not shown), however this phenomenon was not accompanied by the formation of secondary inclusions 72 h post-infection. Further studies will be required to determine if this phenomenon is due to technical limitations of keeping infected EMOs alive for a long period of time or reflective of the response of EMOs to infection and/or inclusion lysis.

The model presented here complements a recently described human fallopian tube organoid model, which has been used to address tubal pathology during chronic *Chlamydia* infection (Kessler et al., 2015, 2019). The fallopian tube organoid and EMO models together provide the ability to observe mechanisms of chlamydial infection in the relevant upper female genital tract tissue. Moreover, generation of human EMOs to model *Chlamydia* infection would further enhance the relevance of the EMO model. Human EMOs have been described (Boretto et al., 2017, 2019; Turco et al., 2017), and the methods developed here for infecting murine EMOs could be easily translatable.

Genetic methods can further develop this model and expand its use in studying pathogenesis of *C. trachomatis*. Genetic manipulation of EMOs using lentiviral vector systems would incorporate the genetic tractability of traditional cancer-derived cell lines. Such systems have already been developed with intestinal organoids and could be adapted for EMOs, as techniques for lentiviral infection are similar to the methods described here for *C. trachomatis* infection (Maru et al., 2016). Another method to improve the genetic tractability of EMOs is the use of genetically modified mice for primary endometrial tissue. Boretto et al. successfully generated EMOs from TdTomato knock-in mice, demonstrating the possibility of using the power of mouse genetics with this system (Boretto et al., 2017).

Together, our results indicate that murine EMOs can be used as a novel system with which to study *C. trachomatis* infection. This model offers a simple system to study direct interactions between *C. trachomatis* and the epithelial cells of the endometrium, providing the relative ease of maintenance and microscopy methods of *in vitro* cell culture, as well as the tissue architecture and relevant cell types of an *in vivo* model.

## Data Availability Statement

The datasets generated for this study are available upon request to the corresponding author.

## Author Contributions

RB performed the experiment. ID supervised the study. RB and ID conceived the experiments, analyzed the data, and wrote the manuscript. MR provided the endometrial tissue. MR, MB, and HV provided expertise and edited the manuscript. All authors contributed to the article and approved the submitted version.

## Conflict of Interest

The authors declare that the research was conducted in the absence of any commercial or financial relationships that could be construed as a potential conflict of interest.
